# BIOLOGICAL MARKERS OF FALLS IN OLDER ADULTS WITH MILD COGNITIVE IMPAIRMENT

**DOI:** 10.1093/geroni/igad104.1478

**Published:** 2023-12-21

**Authors:** Frederico Pieruccini-Faria, Canan Okuyan, Yanina Sarquis-Adamson, Surim Son, Munira Sultana, Manuel Montero-Odasso

**Affiliations:** Gait and Brain Lab/ St. Josephs Hospital/Lawson Research Institute/Parkwood Institute, London, Ontario, Canada; Sakarya University, Academy of Applied Sciences, Antakya, Sakarya, Turkey; Gait and Brain Lab/ St. Josephs Hospital/Lawson Research Institute/Parkwood Institute, London, Ontario, Canada; Western University, London, Ontario, Canada; University of Western Ontario, London, Ontario, Canada; University of Western Ontario, London, Ontario, Canada

## Abstract

Mild Cognitive Impairment (MCI) nearly doubles the risk of falls in older adults. While the association between cognition and falls risk is relatively well understood, no study has explored how biological markers of organic integrity contribute to falls risk in MCI. This study aimed to identify biological markers that could be associated with an increased rate of incidental falls in MCI. A total of 142 participants with MCI (aged 74.3 ± 6.1 years;75% women) with blood tests performed at baseline in Gait and Brain Cohort Study were analyzed. Participants were followed up to 7 years (mean of 28 ± 22.1 months), and falls were reported during the follow-up period(70% fell). Eighteen biological markers extracted from blood serum were dichotomized into low and moderate-to-high levels based on tertile cut-offs. Negative binomial regression model was used to estimate the incident rate ratio (IRR) of falls for biological markers, adjusted for age, sex, global cognition, gait speed, total follow-up duration, and history of falls in the past 12 months at baseline. Results showed that low Parathyroid Hormone (PTH) level (IRR=1.73; 95%CI=1.07, 2.79; p=0.02) and moderate-to-high Interleukine-8 (IL-8) level (IRR= 1.81; 95%CI 1.05-3.14; p=0.03) were associated with an increased rate of falls during the follow-up. Moderate-to-high IL-8 level was also associated with an increased rate of falls with injuries (IRR=2.07; 95%CI=1.05, 4.08; p=0.03). This suggests that in addition to cognitive deficits, impaired calcium metabolism caused by low PTH level and inflammatory processes affecting internal organs may increase falls risk in older adults with MCI.

